# CCL2-Expressing Astrocytes Mediate the Extravasation of T Lymphocytes in the Brain. Evidence from Patients with Glioma and Experimental Models *In Vivo*

**DOI:** 10.1371/journal.pone.0030762

**Published:** 2012-02-02

**Authors:** Maria Angeles Carrillo-de Sauvage, Aurora Gómez, Carmen María Ros, Francisco Ros-Bernal, Eduardo D. Martín, Ana Perez-Vallés, José M. Gallego-Sanchez, Emiliano Fernández-Villalba, Carlos Barcia, Carlos Barcia, Maria-Trinidad Herrero

**Affiliations:** 1 Clinical and Experimental Neuroscience, University of Murcia, Murcia, Spain; 2 Centro de Investigación Biomédica en Red sobre Enfermedades Neurodegenerativas (CIBERNED), School of Medicine, University of Murcia, Murcia, Spain; 3 Instituto de Investigación en Discapacidades Neurológicas (IDINE), University of Castilla-La Mancha, Albacete, Spain; 4 Department of Neurosurgery, Hospital General Universitario de Valencia, Valencia, Spain; 5 Department of Pathology, Hospital General Universitario de Valencia, Valencia, Spain; Institute Biomedical Research August Pi Sunyer (IDIBAPS) - Hospital Clinic of Barcelona, Spain

## Abstract

CCL2 is a chemokine involved in brain inflammation, but the way in which it contributes to the entrance of lymphocytes in the parenchyma is unclear. Imaging of the cell type responsible for this task and details on how the process takes place *in vivo* remain elusive. Herein, we analyze the cell type that overexpresses CCL2 in multiple scenarios of T-cell infiltration in the brain and in three different species. We observe that CCL2^+^ astrocytes play a part in the infiltration of T-cells in the brain and our analysis shows that the contact of T-cells with perivascular astrocytes occurs, suggesting that may be an important event for lymphocyte extravasation.

## Introduction

Lymphocyte infiltration is an important phenomenon in the inflammatory response and is an event involved in many degenerative scenarios of the CNS. Many brain tissue diseases, such as brain tumors and viral or bacterial infections, among many others, show infiltrated T-cell subsets. The effect of such T-cell infiltration may vary in different CNS diseases, but controlling the entrance of blood cells may have important clinical and therapeutic implications. The phenomenon of extravasation is regulated by a cascade of molecular events that involves the adhesion of lymphocytes to the endothelium following their migration into tissue [1], [2], [3]. Selectin-mediated interactions cause lymphocytes to roll over the endothelial cells, at the same time coming into contact different factors. The adhesion molecules involved in the recruitment of lymphocytes into the tissue (such as ICAM-1, VCAM or LFA-1) are well defined but less is known about the factors that trigger the adhesion and direct the extravasation. It has been proposed that chemokines or chemoattractive cytokines trigger the adhesion and migration of lymphocytes either as soluble chemoattractants or as immobilized molecules bound to proteoglycans of the endothelial surface [4]. CCL2 (formerly MCP-1) is one of the most important chemokines involved in the recruitment of blood cells, especially macrophages [5], into the tissues but fewer information is reported about its effect on T lymphocytes. Significantly, CCL2 induces the formation of the uropod in T-cells [1], a cellular formation crucial for the cell's entrance into the parenchyma. However, identification of the brain cells in charge of this phase of extravasation in the CNS and data on how the process takes place remain elusive. In the present work we analyze the cellular expression of CCL2 in three independent scenarios of T-cell infiltration in the brain *in vivo*. We observe that the perivascular astrocytes highly express CCL2, independently of the species and the inflammatory situation, facilitating the entrance of lymphocytes into the brain. CCL2-expressing astrocytes are specifically localized in the T-cell-infiltration areas independently of the inflammatory scenario. Importantly, blocking CCL2 attenuates the penetration of T-cell infiltration in the brain, which suggests that CCL2 produced by reactive astrocytes at the site of inflammation is responsible for T-cell extravasation. On the other hand, the physical contact between CCL2^+^ astrocytes and CD3^+^ T-cells takes place at perivascular areas of the brain and we hypothesized that this phenomenon may be important for lymphocyte internalization.

## Materials and Methods

### Patients and Samples

Fourteen patients from the General Hospital of Valencia (Spain) were diagnosed with glioma of different degrees and were cited for intracranial surgery. The fourteen cases showed the typical features of astrocytoma, with the classic clinical evolution, neuroimaging, spectroscopy, and anatomo-pathological examination (**Figure S1**). The study was carried out according to the approved protocols of all the institutions involved (General Hospital of Valencia and University of Murcia). The approval from the IRB of the University of Murcia was received regarding the project (RYC-2010-06729) and a written informed consent was obtained from all participants involved in the study (General Hospital Permits # 07B0002707-09B0010238; University of Murcia Project ID: RYC-2010-06729). The brain tumors presented characteristic glioma morphology. Tumors were excised and processed with a previously published protocol [6].

### Adenovirally injected monkeys

All primate samples were collected for previously published studies [7]. In the present work, we analyzed the brain tissue sections from seven adult (either male or female) cynomolgus monkeys (*Macaca fascicularis*) (5 kg body weight) that were processed in preceding studies [7]. Briefly, the animals were injected bilaterally with 1×10^7^ infective units of Ad-mCMV-βgal adenovirus (Kindly provided by Dr. P.R. Lowenstein and Dr. M.G. Castro) in six brain coordinates, including cortex and white matter and the tissue was processed as described previously [7]. In all studies involving non-human primates, all necessary steps were taken to ameliorate any suffering or pain. The referenced study was carried out according to the approved protocols of the University of Murcia (Project ID: FEDER FD1/97-1931) and all the experiments were conducted in accordance with the Guidelines of the European Convention for the protection of Vertebrate Animals used for Experimental and other scientific purposes of the Council of Europe (n° 123, June 15^th^ 2006).

### Mice

Twenty-seven adult male C57BL6 mice were used to test the role of CCL2 in the infiltration of lymphocytes in the brain. Fourteen mice were used in a first experiment to analyze the correlation between the infiltration of T-cells and the expression of CCL2 cells after LPS injection. On day 0, mice were anesthetized using ketamine (50 mg/kg) and medetomidine (1 mg/kg) and either 2 mg/ml of LPS (n = 7) or saline (n = 7) in 1 ml of volume was injected into the right striatum as previously described [8]. Four days were allowed to pass waiting for the immune response to be stimulated. Then, animals were sacrificed on day 7, at the peak of the potential T-cell response as has been previously described [9]. On the other hand, in order to know the effect of CCL2 in the lymphocyte infiltration, another experiment was performed injecting goat anti-mouse CCL2 neutralizing antibody (R&D Systems) directly into the brain parenchyma. Mice were anesthetized and placed in the stereotactic frame and an injecting guide cannula (Plastics One, C315G-SPC, Roanoke, VA, USA) was placed in the right striatum in the same coordinates indicated above. The guide cannula was then protected with a dummy cannula (Plastics ONE, C315CD-SPC) following the instructions of the manufacturers. After surgery, animals were left in their cage for 15 days in order to eliminate/diminish the inflammation caused by the intra-parenchymal placement of the cannula. After this time, animals received an injection of LPS (2 mg/ml) using an internal cannula (Plastics ONE, C3151-SPC), through the previously implanted guided cannula, and four days later they received, through the same cannula and with the help of a microinjector, either an intraparenchymal injection of goat anti-mouse CCL2 neutralizing antibodies (0.1 µg/µl, n = 3 or 1 µg/µl, n = 5) or the purified isotype immunoglobulin (Goat IgG, 0.1 µg/µl) on day 4^th^ and 6^th^. Seven days after the LPS injection animals were anesthetized and sacrificed and processed as described below.

Mice were anesthetized with an overdose of ketamine/xylacine for perfusion-fixation (with PBS followed by 4% PFA). Brain tissue was then removed and post-fixed in PFA for 48 hours. The study was carried out according to the approved protocols of the University of Murcia (Project ID: FIS/PI10/02827). All the experiments were conducted in accordance with the Helsinki Declaration, the Guide for the Care and Use of Laboratory Animals (NIH Guide, revised 1996), the Guidelines of the Europeanonvention for the protection of Vertebrate Animals used for Experimental and other scientific purposes of the Council of Europe (no 123, June 15th, 2006) and the European Communities Council Directive 2010/63/ECC.

### Immunocytochemical procedures

Brain tumor sections (60 µm) were selected according to the anatomopathological criteria (**Figure S1**), cut serially through the entire sample, and immunofluorescence or diaminobenzidine (DAB) detection was performed as described previously [6]. Coronal monkey brain sections (50 µm) were cut serially through the entire brain, and DAB detection or immunofluorescence was performed as described previously [7]. In addition, hematoxilin staining was performed in some of the sections to confirm the localization of tumorigenic areas and blood vessels (BV). For human or monkey tissue the following primary antibodies were used: anti-human CCL2 (MCP-1) (1∶40, mouse IgG2b; R&D), anti-human CD3 (1∶100, Rabbit or 1∶50, Mouse, IgG1; Dako), anti-human CD20 (1∶500, mouse, IgG2a; Dako), anti-GFAP (1∶500, Rabbit; Chemicon), anti-LFA-1 (1∶50, Rabbit; Abcam), anti-human ICAM-1 (1∶100, Mouse, IgG1; Sigma-Aldrich), anti-human VCAM-1 (1∶50, mouse, IgG1k; Dako), anti-CCR2 (1∶200, goat; Abcam) and Collagen-IV (1∶500, Rabbit; Abcam). Mouse brains were sectioned in 30 µm serial sections using a vibratome (Leica), and were stained for immunofluorescence or DAB detection using previously published protocols [8]. The following antibodies were used: anti-mouse CCL2 (1∶200, Rabbit, Serotec), anti-mouse CD4 (1∶750, Rat; Serotec), anti-mouse CD8 (1∶750, Rat; Serotec), anti-mouse CD3 (1∶750, Hamster, Serotec) and anti-GFAP (1∶500; mouse, Chemicon, Millipore).

### Quantification and sterological analysis

The number of labeled cells was quantified as previously described [10]. CCL2^+^ cells and CD3+ T-cells were quantified in all species studied. In mice, the number of CD4^+^ and CD8^+^ T-cells was also counted. Correlations between the number of CCL2^+^ cells and the number of CD3^+^ cells were calculated from the data obtained from the quantifications of DAB^+^ immunostained sections. To obtain the data for the correlations, two independents stereological quantifications of CD3 and CCL2 were performed in serial adjacent sections in all specimens. Results were expressed as the mean ± SEM.

### Confocal imaging

Brain sections from human biopsies, monkey and mouse brain were examined by confocal microscopy as described previously [7]. Images are shown as the transparency of the stack of images or showing a single 0.5 µm optical section.

### Statistical analysis

Data are expressed as mean ± SEM. Statistical analysis was performed using student's t-test or one-way ANOVA test following a posthoc analysis. The null hypothesis was rejected for an α risk equal to 5%.

## Results

**Figure 1:**
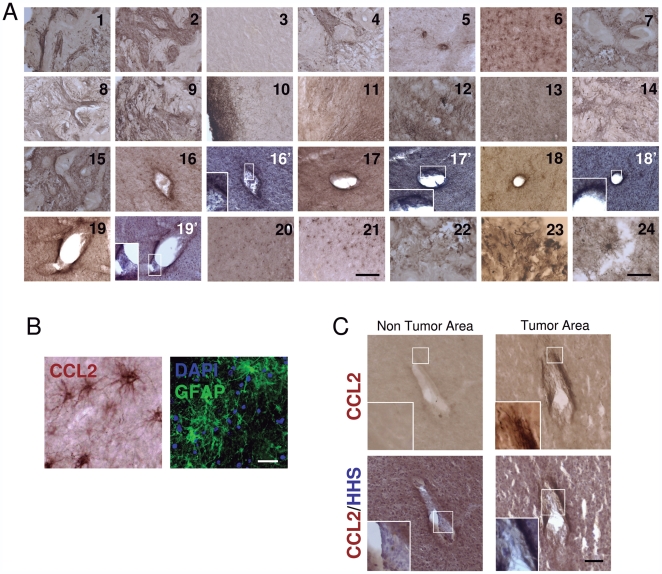
Human glioma shows CCL2^+^ cells. (A) The 14 cases of glioma analyzed show CCL2 expression in the tumorigenic areas. Samples of gliomas were immunostained to detect the expression of CCL2. All 14 cases analyzed showed expression of CCL2 in the neoplasic areas. CCL2-expressing cells are localized in the brain parenchyma itself and their number and intensity increase towards the necrotic areas. CCL2-expressing cells can also be seen around BVs (16–19). In addition, counterstaining with hematoxilin is also shown at BV levels (16′–19′) to corroborate the presence of endothelial nuclei. Insert show a detail of the endothelial nuclei. Scale bar; 1–21: 400 µm, 22–24: 60 µm. (B) Tumor cells present high immunoreactivity for GFAP in tumor areas, demonstrating the typical astrocytic cell type. CCL2^+^ cells show a characteristic astrocytic morphology. Scale bar: 30 µm. (C) BVs in, or close to the putative tumorigenic areas show high immunoreactivity for CCL2 in contrast to normal tissue. Additionally, counterstaining with hematoxilin (HHS) is also shown at the same levels. The insert shows a detail of the endothelial nuclei. Scale bar: 100 µm.

**Figure 2:**
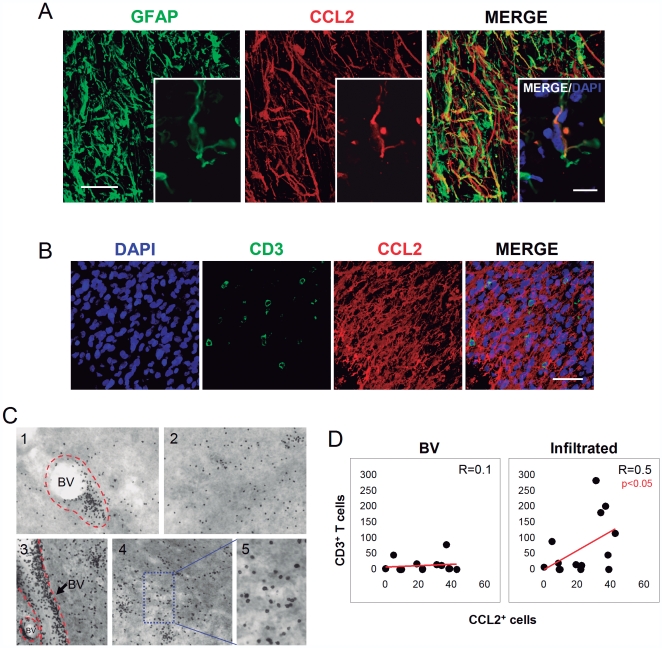
CCL2^+^ cells are GFAP^+^ astrocytes in human glioma. (A) Confocal images show co-localization of CCL2-expressing cells (red) with GFAP (green) in glioma. Insert shows a detail of a single CCL2-expressing cell (red) co-localizing with GFAP (green) and counterstained with DAPI (blue). Scale bar: 30 µm. (B) Areas of infiltration of CD3^+^ T-cells (green) coincide with the areas of CCL2-expressing cells (red) in glioma. Confocal images show a tumorigenic area, infiltrated with CD3^+^ T-cells (green) in an area with numerous CCL2-expressing cells (red). The immunostaining was combined with a counterstaining with DAPI to detect the nucleus (blue). Scale bar: 50 µm. (C) Examples of characteristic CD3 infiltration in samples of glioma. CD3^+^ T-cells can be observed grouped in BVs (BV, limited by broken red line in 1 and 3) but also infiltrated in the parenchyma (2, 4, blue insert magnified in 5). (D) CCL2 expression correlates with the infiltration of T-cells in the tumor areas. The quantification in serial sections of the number of T-cells, either infiltrated or located in the BV lumen, revealed that the level of infiltration of CD3^+^ T-cells in the parenchyma (green) is positively correlated with the level of CCL2 expression (BV; BV lumen).

All biopsies of patients with glioma presented high expression of CCL2 in tumorigenic areas (**Figure 1A**). Importantly, all the cases studied showed CCL2 immunoreactive cells, while the shape of the areas of immunoreactivity differed according to the structure of the tumor (**Figure 1A** **, images 1–24**). Areas far from the tumorigenic center, putatively healthy tissue, did not show CCL2 immunoreactivity (**Figure 1A** **, image 3**) and a gradient of immunoreactivity from non tumorigenic to tumorigenic areas could be clearly seen in most cases (**Figure 1A** **, image 10**). CCL2 was especially high around BVs' lumen (**Figure 1A** **, images 16–19 and 16′–19′**) and single cells showed star shape structures (**Figure 1A** **, images 20–24**) similar to astrocyte-specific GFAP staining (**Figure 1B**). BVs, inside and close to tumorigenic areas, strongly expressed CCL2, but did not do so outside the tumorigenic areas (**Figure 1C**). Importantly, confocal co-localization analysis revealed that astrocytes were the CCL2-expressing cells in the tumor area (**Figure 2A**).

These CCL2^+^ areas were seen to present the specific infiltration of CD3^+^ T-cells in brain tumors (**Figure 2B**). However, in order to understand the role of CCL2 in the specific extravasation of CD3^+^ lymphocytes, the correlation between the number of tumor infiltrating CD3^+^ T-cells and the number of expressing CCL2 cells in the glioma samples was calculated. Importantly, we divided the T-cells into two different populations: T-cells inside the BV lumen and those that were extravasated (**Figure 2C**). Interestingly, we found that the amount of T-cells circulating in the BV lumen was independent of CCL2 expression and no correlation was observed (**Figure 2D**). Conversely, a significant correlation was seen between the expression of CCL2 and the infiltration of T-cells in the tumorigenic parenchyma (**Figure 2D**). These results suggest that the presence of CCL2^+^ cells may be important for the extravasation of lymphocytes, although a chemotactic effect of secreted CCL2 into the blood circulation does not seem to contribute to this phenomenon.

**Figure 3:**
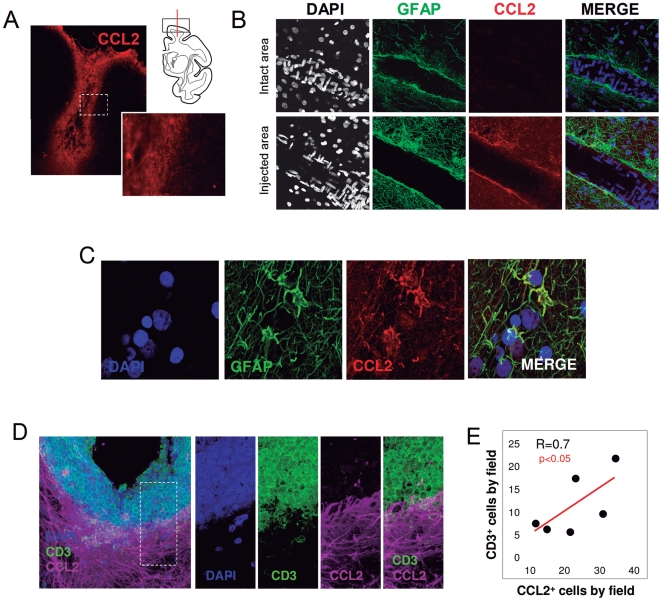
CCL2^+^ astrocytes are expressed in T-cell infiltration areas in monkey brain. (A) Immunofluorescence shows the specific CCL2 expression located at the area of adenoviral injection in the macaque brain. Drawing on the right shows the anatomical location of the stereotaxic injection in the brain cortex. (B) Confocal images of BVs in an intact area and an injected area of the monkey brain. Perivascular astrocytes marked with GFAP (green) show CCL2 expression (red) in the adenoviral-injected areas. DAPI (blue) was used as a nuclear counterstaining to show the peculiar disposition of endothelial nuclei. (C) CCL2 is expressed by astrocytes in inflamed monkey brain. The staining of CCL2 (red) colocalizes with the astrocyte marker GFAP (green) in monkey cortex in the adenovirally injected area. (D) Three dimensional transparency of an area of CD3^+^ T-cells (green) infiltration shows specific CCL2 expression (magenta) in the brain of adenoviral injected monkey. DAPI was used as a counterstaining. Details of the insert are shown in the images on the right. (E) The stereological quantification of serial adjacent sections demonstrated that the number of infiltrated CD3^+^ cells correlates with CCL2^+^ cells in adenoviral injected areas in monkey brain.

To investigate the role and cell type of CCL2 in other inflammatory scenarios two animal experimental models of inflammation and T-cell infiltration were studied, first using brain tissue from monkeys that had been infected stereotactically in the cerebral cortex with artificially engineered adenoviral vectors. Previous published analyses have shown that adenoviral injections in the CNS elicit a specific infiltration of lymphocytes restricted to the injection areas [7]. We analyzed the presence of lymphocytes in the areas of injection, which were seen to express ICAM-1, VCAM (**Figures S2 and S3**) in contrast with intact areas (**Figure S4**). In addition, infiltrating lymphocytes express LFA-1 in the extravasation areas (**Figure S5**). Moreover, these areas of viral injection showed a particular expression of CCL2 limited to the injection area (**Figure 3A**). Importantly, confocal analysis of the infiltrated areas showed highly immunoreactive CCL2-expressing cells around BVs that were not present in intact (non-injected) areas (**Figure 3B, C** **and Figure S6**). In addition, detailed analysis of the virally injected areas demonstrated co-localization of CCL2^+^ and GFAP^+^ cells (**Figure 3D**). Importantly, the areas of CD3^+^ T-cell infiltration show CCL2 expressing cells (**Figure 3E**) and a positive correlation was found between the number of infiltrated CD3^+^ T-cells and the number of CCL2^+^ cells in the adenoviral injected areas of monkey brain.

**Figure 4:**
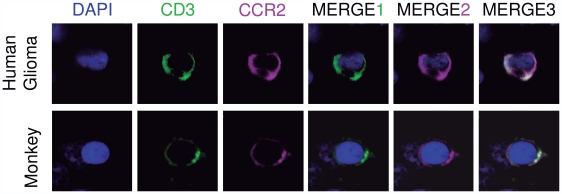
T cells express CCL2 receptor. Expression of CCR2 in T-cells in human glioma (top panel) and monkey brain (bottom panel). CD3^+^ T-cells (green) express CCR2 in their surface (magenta). Nucleus is stained with DAPI (blue).

Since CD3^+^ T-cells seem to respond to CCL2 in the inflamed parenchyma in the human glioma and monkey brain, we analyze their ability to express the CCL2 receptor (CCR2) in their cell surface. We immuno-stained the glioma samples and monkey brain sections with an antibody against CCR2 combined with CD3 T-cell marker. Importantly, we observed that CD3^+^ T-cells are able to express CCR2 in their surface (**Figure 4**), which makes possible the interaction ligand-receptor in the extravasation phenomenon. In addition, to rule out whether the immunopositivity for CCL2 in astrocytes might reflect the binding to CCR2, we immunostained the sections with an antibody against CCR2 combined with the astrocytic marker GFAP. We observe that CCR2 is not expressed in astrocytes (**Figure S7**) suggesting that the immunopositivity for CCL2 in astrocytes does not correspond to ligand binding to its receptor.

**Figure 5:**
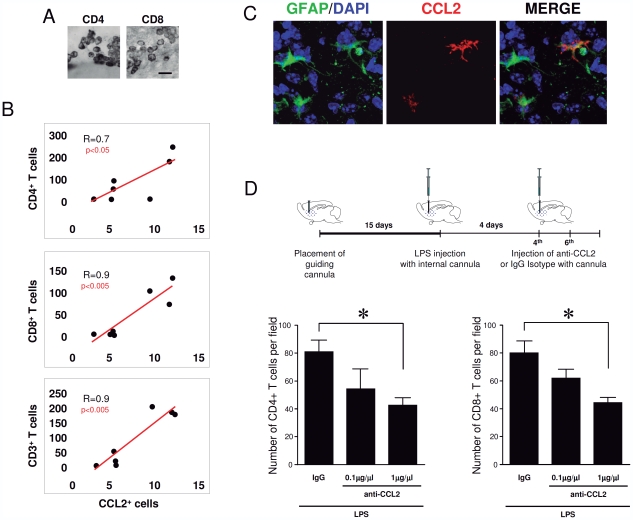
Intracerebral blocking of CCL2 attenuates the LPS-mediated T-cell infiltration. (A) Detail of CD4^+^ and CD8^+^ T-cell immunostaining in the striatum of LPS injected mice. Scale bar: 20 µm. (B) The number of infiltrated T-cells (CD4^+^, CD8^+^ and CD3^+^ T-cells) correlates with the number of CCL2^+^ cells in the areas of LPS injection. (C) Intraparenchymal injection of anti-CCL2 antibodies attenuates the LPS-induced infiltration of lymphocytes in the mouse brain parenchyma. The diagram on the top shows the arrangement of the experiment. Graphs show the density of CD8 and CD4 T-cells in the brain parenchyma surrounding the injection site. (D) Astrocytes express CCL2 after LPS injection in mouse brain. Confocal images of the injected areas show the co-localization of GFAP^+^ astrocytes (green) and CCL2 (red) in mouse brain. DAPI (blue) was used as a nuclear counterstaing. * p<0.05 ANOVA-test.

In order to further understand the functionality of CCL2 in mediating T-cell infiltration into the brain parenchyma, we induced brain inflammation with the stereotaxic injection of LPS in the cortex and striatum of mice. After the LPS injection, we observed a clear T-cell infiltration in the brain parenchyma evidenced by the expression of T-cell markers, CD3, CD4 and CD8 at the injection area (**Figure 5A and B**). The specific infiltration of T-cells in the brain after LPS was observed concomitantly with an increase in CCL2 expression limited to the injected areas. In fact, we observed a significant positive correlation between the number of infiltrated T-cells and the number of CCL2^+^ cells (**Figure 5B** **and Figure S8**). Detailed confocal co-localization analysis demonstrated that the CCL2^+^ cells were also astrocytes in LPS-induced infiltration in mice (**Figure 5C**), similar to the situation observed in human gliomas and monkey brain. These results suggest that CCL2-expressing astrocytes may contribute to the T-cell infiltration into the brain parenchyma. To better understand the intraparenchymal effect of CCL2, we set up an experiment using an anti-CCL2 neutralizing antibody, injected directly into the brain through an intracranial cannula as described in detail in the methods section. Briefly, after placing a guiding cannula in the mouse brain parenchyma, we waited 15 days to allow the restoration of the inflammation caused by the surgery (see diagram in **Figure 5D**). Then, we injected LPS through the implanted guiding cannula to induce inflammation, and after 4 days we injected either anti-CCL2 neutralizing antibody or the specific immunoglobulin isotype as a control. The histological analysis of the infiltration of CD4 and CD8 T-cells revealed that the injection of anti-CCL2 reduces significantly the infiltration of CD4^+^ and CD8^+^ lymphocytes (**Figure 5D**). This infiltration is lowered with 0.1 µg/µl but it is significantly diminished with 1 µg/µl which suggests a dose dependent effect of the CCL2 neutralizing antibody administration. Taken together, these results show that CCL2 functionally mediates the extravasation of T-cells in mouse brain upon inflammation.

**Figure 6:**
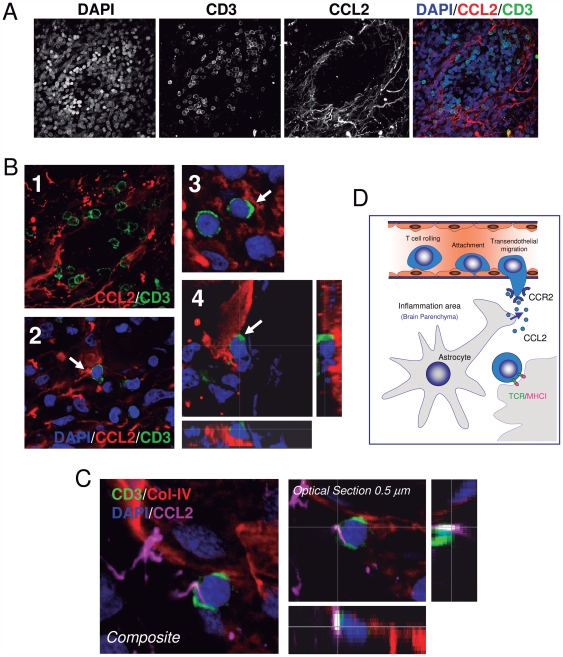
T-cells come into contact with CCL2^+^ perivascular astrocytes in the areas of infiltration. (A) CCL2 is highly expressed in perivascular astrocytes where T-cells infiltrate the brain tumor. Detailed confocal analysis of samples of gliomas revealed that tumorigenic areas show CCL2-expressing astrocytes (red) with infiltration of CD3^+^ T-cells (green). Nuclei are stained with DAPI (blue). (B) Infiltration of T-cells occurs throughout multiple anatomical contacts between T-cells and CCL2-expressing astrocytes. Image B1 shows numerous CD3^+^ T-cells (green) establishing specific contacts with CCL2^+^ cells (red). In pictures B2, 3 and 4, a detail of T-cells (green) in contact with CCL2 perivascular astrocytes. Image B4 shows a specific detail of a CD3/CCL2 contact on the xy axis and the two lateral views on the z axis. (C) Confocal analysis of a CD3^+^ T-cell coming into contact with a CCL2^+^ cell located at the perivascular area of adenoviral injected monkey brain. The three dimensional transparency (Composite) of a stack of images shows a CD3^+^ T-cell (green), in close apposition to the endothelium, marked with Col-IV (red), and contacting a CCL2^+^ cell (magenta). A detail of the CD3-CCL2 contact is also shown in a 0.5 µm optical section on the xy axis and the two lateral views on the z axis. (D) Diagram showing how T-cells may come into contact with CCL2-expressing astrocytes in the edge of BVs suggesting that CCL2^+^ astrocytes contribute to the extravasation of T-cells in the brain parenchyma.

Our results suggest that CCL2-expressing astrocytes may contribute to the specific extravasation of lymphocytes. Since there is no correlation between CCL2^+^ astrocytes and T-cell number in the lumen of the BV at the sites of inflammation, a chemotactic effect of secreted CCL2 does not seem to contribute to this phenomenon. Alternatively, CCL2-expressing astrocytes might need to physically interact with T-cells in order to mediate lymphocyte extravasation. To explore this possibility a detailed high-resolution confocal analysis of the anatomical location of infiltrating T-cells in the BVs and in the brain parenchyma of inflammatory areas was carried out. Importantly, specific contacts between CCL2^+^ cells and CD3^+^ lymphocytes were found in the areas of infiltration (**Figure 6A and B**) and at the periphery of BVs (**Figure 6C**), suggesting that the specific attachment of T-cells to the perivascular astrocytes may be a crucial event in the extravasation of T-cells in the inflamed brain (**Figure 6D**).

## Discussion

In the present work, we show that, independently of the species and of the CNS inflammatory scenario, the extravasation of lymphocytes is mediated by CCL2-expressing astrocytes. In the three inflammatory situations illustrated, we found a specific increase of CCL2^+^ cells in the affected areas with a concomitant T-cell infiltration. Importantly, our confocal images demonstrate that perivascular astrocytes are responsible for the expression of CCL2 in the three analyzed T-cell-infiltration situations in the brain, in the three different species and with multiple antibodies specific for CCL2. Our results demonstrate that the expression of CCL2 by astrocytes contributes to the entrance of lymphocytes in the brain parenchyma. In the experiments performed in mice, the blocking of CCL2 by a specific neutralizing antibody attenuated the infiltration of lymphocytes which suggests that CCL2, expressed by astrocytes, contributes to the internalization of the T-cells into the parenchyma. Furthermore, the fact that the level of CCL2 expression correlates with the T-cell infiltration proper and not with the number of lymphocytes circulating in the BVs, strongly suggests that the presence of CCL2^+^ astrocytes contributes to the process of lymphocyte extravasation and play a part in the cellular entrance. In addition, our data demonstrate that the anatomical contact between lymphocytes and CCL2^+^ astrocytes takes place in the internal wall of BV and we suggest that may have a contributing role in the actual infiltration to the brain parenchyma.

Our results also narrow down the controversy regarding the cell type that is responsible of CCL2 expression in the brain. Previous studies in biopsies of patients with multiple sclerosis and after brain injury in mice have suggested that CCL2 is expressed by astrocytes but no co-localization analysis of the proteins was conducted [11], [12]. In the present work, we show that CCL2 co-localizes with GFAP in three different species, with two different antibodies, and in three different situations of inflammatory-mediated lymphocyte infiltration.

Importantly, in our study we hypothesize that the anatomical apposition of T-cells and CCL2^+^ astrocytes may be an important event in the internalization of lymphocytes in the brain parenchyma. The CCL2 function was thought to be mainly chemotactic, but our results suggest that the physical contact between T-cells and astrocytes may also contribute to the actual internalization into the brain parenchyma proper, probably after the two phenomena of rolling and adhesion, which is coherent with the induction of the uropod formation by CCL2 and penetration through the endothelium, contributing to diapedesis (**Figure 6D**).

From a therapeutic point of view, the manipulation of CCL2 may have beneficial effect in neurodegenerative diseases. CCL2 expression has been related with the aggressiveness of the glioma [13] and, in fact, the use of CCL2 neutralizing antibodies has been suggested as a possible strategy for their treatment [14]. However, research into CCL2-mediated infiltration has mainly focused on the role of tumor-infiltrated macrophages but not other cell types [15]. Our present work suggests that, like macrophages, the CCL2 may also mediate lymphocyte infiltration in the glioma, and contribute to the level of aggressiveness. In fact, in a previous report, we demonstrated that T-cells infiltrate gliomas to contact tumorigenic cells although a very low percentage shows cytolitic features [6]. This suggests that the most tumor-infiltrated lymphocytes are regulatory, reducing the activity of the cytolitic cells and allowing the tumor to grow. Therefore, the manipulation of CCL2 may be one of the particular therapeutic targets to control the infiltration of lymphocytes in brain tumors. Accordingly, in other neuroinflammatory processes, such as acute brain trauma, stroke, as well as during chronic affections like multiple sclerosis or Alzheimer's disease, prolonged and sustained inflammation mediated by CCL2 may have cytotoxic effects, aggravating the incidence and the severity of the disease [16]. Therefore, targeting CCL2, more specifically in perivascular astrocytes, in CNS inflammatory scenarios may be particularly important from a therapeutic perspective.

## Supporting Information

Figure S1 **Tumors show typical anatomopathological glioma characteristics.** (A) Area of a sample of glioma with hypercellular appearance with hemorragic areas. Insert shows details of the marked hypercellularity. (B) Sample of a case of glioma showing pleomorphic cells, aberrant mitosis and areas of necrosis. Insert shows a detail of the pleomorhic cells and mitosis. Necrosis area is indicated with an asterisk (*). (C) Sample of glioma showing glomeruloid vessels (*), areas of necrosis (arrow) and gemistocytic cells (Insert). (D) Sample of glioma showing glomeruloid vessels (*). Insert shows a detail of a glomeruloid vessel. Scale bars are indicated in each insert.
(TIF)

Figure S2 **ICAM-1 expression in BVs in areas of infiltration after viral injection in monkey brain.** Top panel shows over-expression of ICAM-1 (red) in perivascular areas, where T-cells (green) infiltrate the brain parenchyma. DAPI was used to stain the nuclei (blue). Bottom panel shows different putative steps (from 1 to 6) of T-cell infiltration in the brain. (1, 2) T-cells (green) rolling through the ICAM-1^+^ endothelial wall (red). Picture 2 shows a rolling T-cell displaying a putative uropod (white arrow). (3, 4) T-cells (green) in the adhesion process. (5, 6) T-cells in the extravasation process.
(TIF)

Figure S3 **VCAM-1 expression in BVs in areas of infiltration after viral injection in monkey brain.** Top panel shows over-expression of VCAM-1 (red) in perivascular areas, where T-cells (green) infiltrate in the brain parenchyma. DAPI was used to stain the nuclei (blue). Bottom panel show a detail of the area of infiltration.
(TIF)

Figure S4 **Intact areas do not express ICAM-1 or VCAM-1 in monkey brain.** Confocal images of BV in intact areas of the macaque brain. ICAM-1 and VCAM-1 are not over-expressed in BV and no CD3^+^ T-cell infiltration is seen in non injected areas.
(TIF)

Figure S5 **LFA-1 expression in lymphocytes in areas of infiltration after viral injection in monkey brain.** Top panel shows over-expression of LFA-1 (magenta) in perivascular areas, where T-cells (green) and B cells (red) infiltrate the brain parenchyma. DAPI was used to stain the nuclei (blue). Bottom panel shows a detail of the area of infiltration of T and B lymphocytes co-localizing with LFA-1.
(TIF)

Figure S6 **Staining of collagen-IV was performed to unequivocally delineate blood vessels shown in** **figure 3B** **.** Confocal images show BVs in monkey brain sections stained with DAPI (white) and collagen-IV (green). In the merged images DAPI is shown in blue.
(TIF)

Figure S7 **CCR2 is not expressed in GFAP^+^ astrocytes.** Immuno-staining of CCR2 in monkey brain and samples of glioma combined with the astrocytic marker GFAP. (A) The areas of adenoviral injection in monkey brain show CCR2^+^ cells (magenta) and do not co-localize with GFAP marker (green). DAPI was used as a counterstaining (blue). Insert shows contact between a CCR2^+^ cell and a GFAP^+^ astrocyte. (B) Samples of glioma (GBM1, GBM2 and GBM3) show CCR2^+^ cells (magenta) and do not co-localize with GFAP^+^ astrocytes. DAPI was used as a counterstaining (blue). Scale bars: 50 µm.
(TIF)

Figure S8 **Increase in the number of CCL2^+^ cells in the mouse brain after LPS intrastriatal injection.** (A) Representative picture of CCL2^+^ cells in the mouse striatum after LPS injection compared to a saline injection. (B) Quantification of CCL2^+^ cells in the injected striatum in a group of mice injected with saline compared with a group of mice injected with LPS. A dramatic increase of CCL2^+^ cells can be observed in the LPS injected mice. *p<0.05 Student t-test.
(TIF)
