# Supplementary figures and images for: CCL2-Expressing Astrocytes Mediate the Extravasation of T Lymphocytes in the Brain. Evidence from Patients with Glioma and Experimental Models In Vivo
Source: PLoS One. 2012 Feb 2;7(2):e30762. doi: 10.1371/journal.pone.0030762 (PMC3271104; doi:10.1371/journal.pone.0030762)

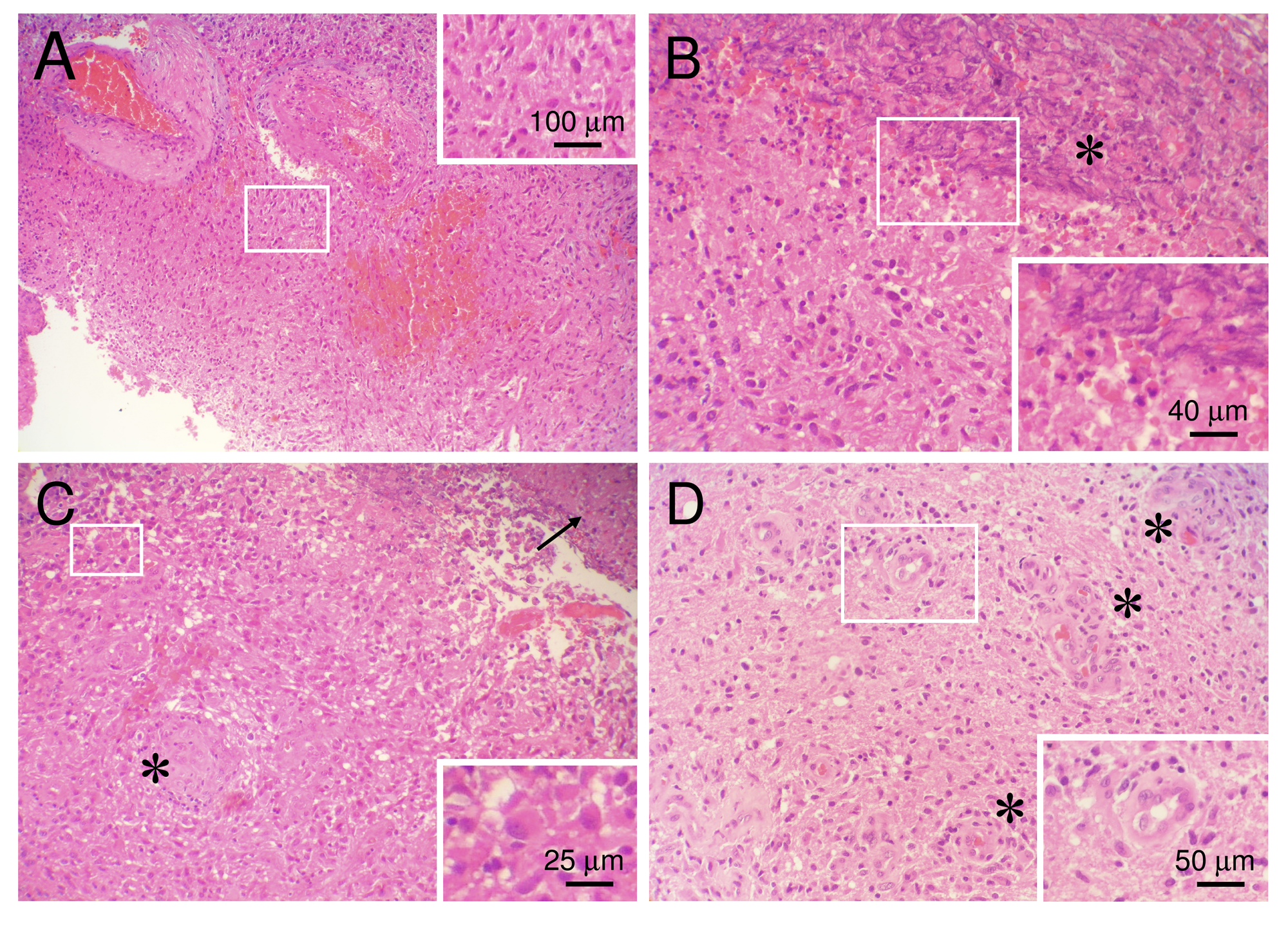

Supplement: Figure S1 — Tumors show typical anatomopathological glioma characteristics. (A) Area of a sample of glioma with hypercellular appearance with hemorragic areas. Insert shows details of the marked hypercellularity. (B) Sample of a case of glioma showing pleomorphic cells, aberrant mitosis and areas of necrosis. Insert shows a detail of the pleomorhic cells and mitosis. Necrosis area is indicated with an asterisk (*). (C) Sample of glioma showing glomeruloid vessels (*), areas of necrosis (arrow) and gemistocytic cells (Insert). (D) Sample of glioma showing glomeruloid vessels (*). Insert shows a detail of a glomeruloid vessel. Scale bars are indicated in each insert. (TIF) [file pone.0030762.s001.tif]

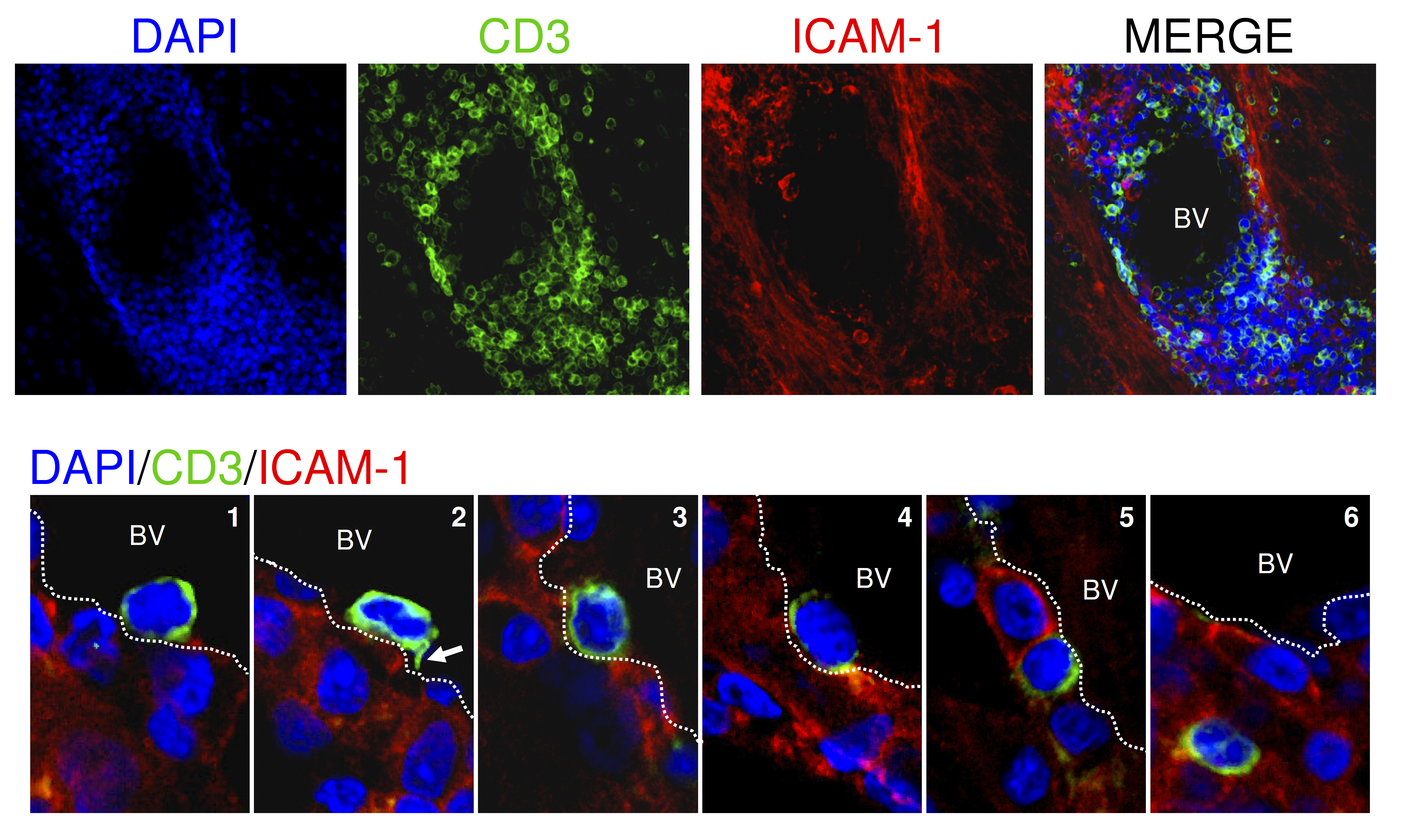

Supplement: Figure S2 — ICAM-1 expression in BVs in areas of infiltration after viral injection in monkey brain. Top panel shows over-expression of ICAM-1 (red) in perivascular areas, where T-cells (green) infiltrate the brain parenchyma. DAPI was used to stain the nuclei (blue). Bottom panel shows different putative steps (from 1 to 6) of T-cell infiltration in the brain. (1, 2) T-cells (green) rolling through the ICAM-1+ endothelial wall (red). Picture 2 shows a rolling T-cell displaying a putative uropod (white arrow). (3, 4) T-cells (green) in the adhesion process. (5, 6) T-cells in the extravasation process. (TIF) [file pone.0030762.s002.tif]

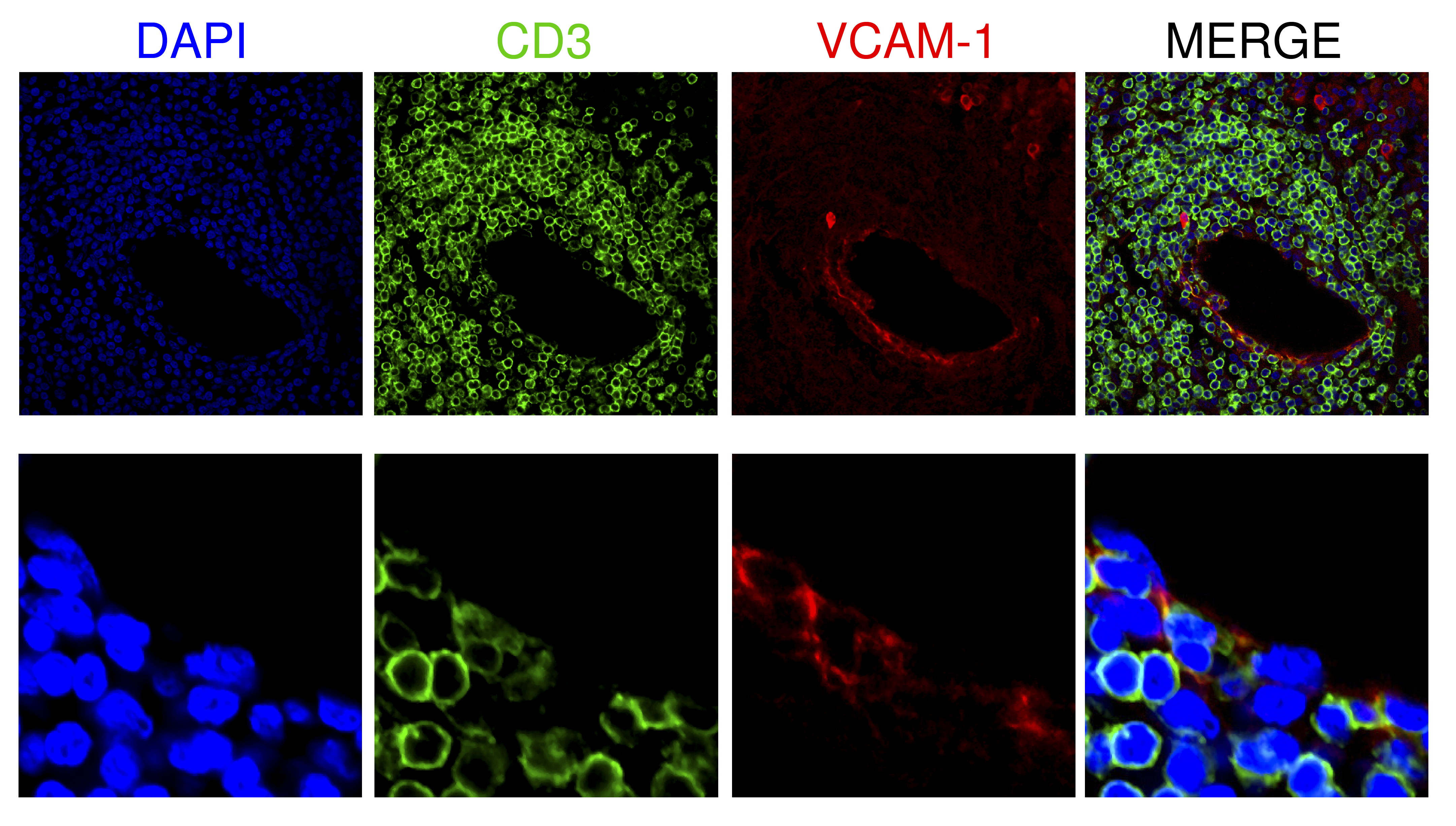

Supplement: Figure S3 — VCAM-1 expression in BVs in areas of infiltration after viral injection in monkey brain. Top panel shows over-expression of VCAM-1 (red) in perivascular areas, where T-cells (green) infiltrate in the brain parenchyma. DAPI was used to stain the nuclei (blue). Bottom panel show a detail of the area of infiltration. (TIF) [file pone.0030762.s003.tif]

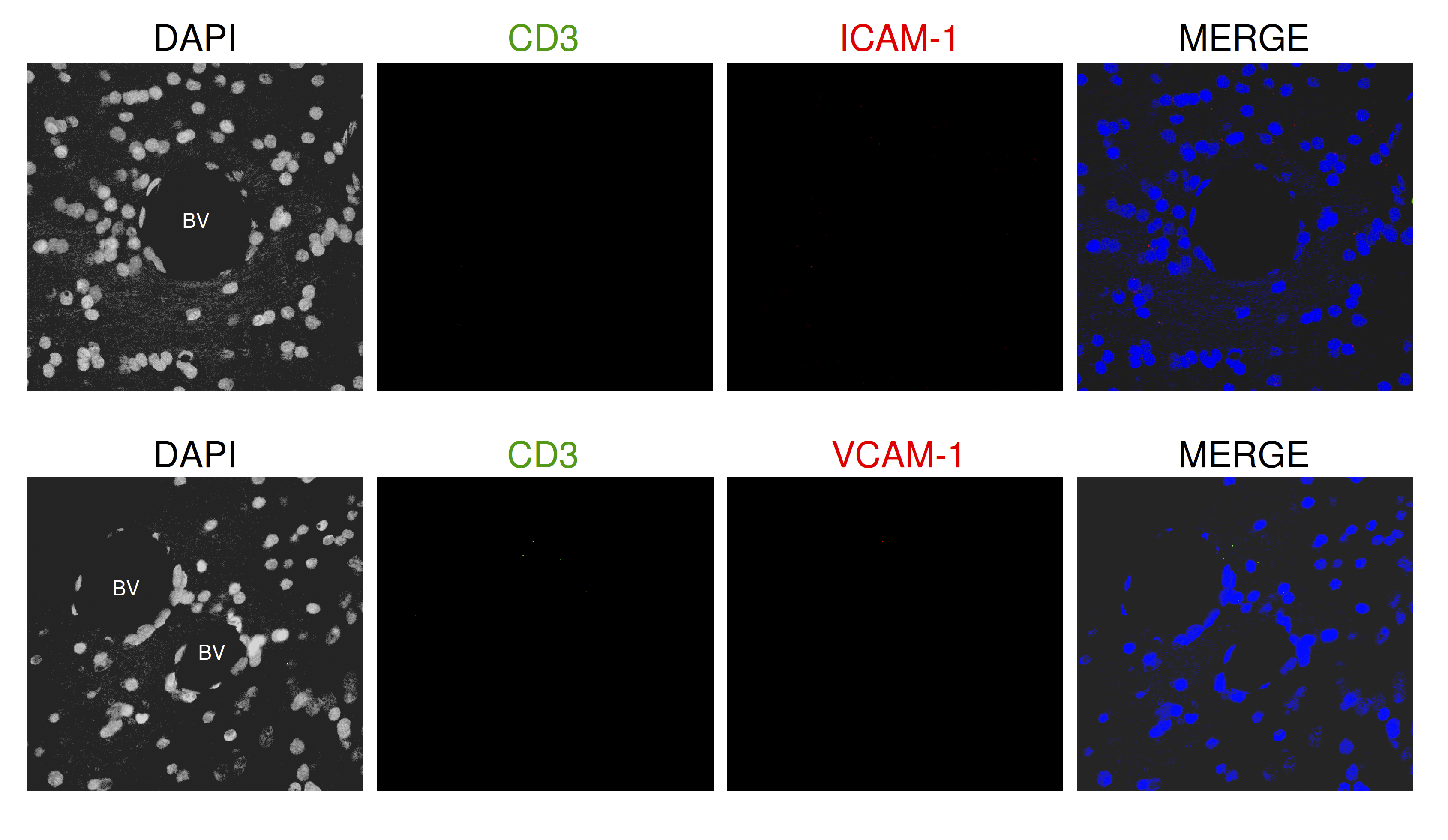

Supplement: Figure S4 — Intact areas do not express ICAM-1 or VCAM-1 in monkey brain. Confocal images of BV in intact areas of the macaque brain. ICAM-1 and VCAM-1 are not over-expressed in BV and no CD3+ T-cell infiltration is seen in non injected areas. (TIF) [file pone.0030762.s004.tif]

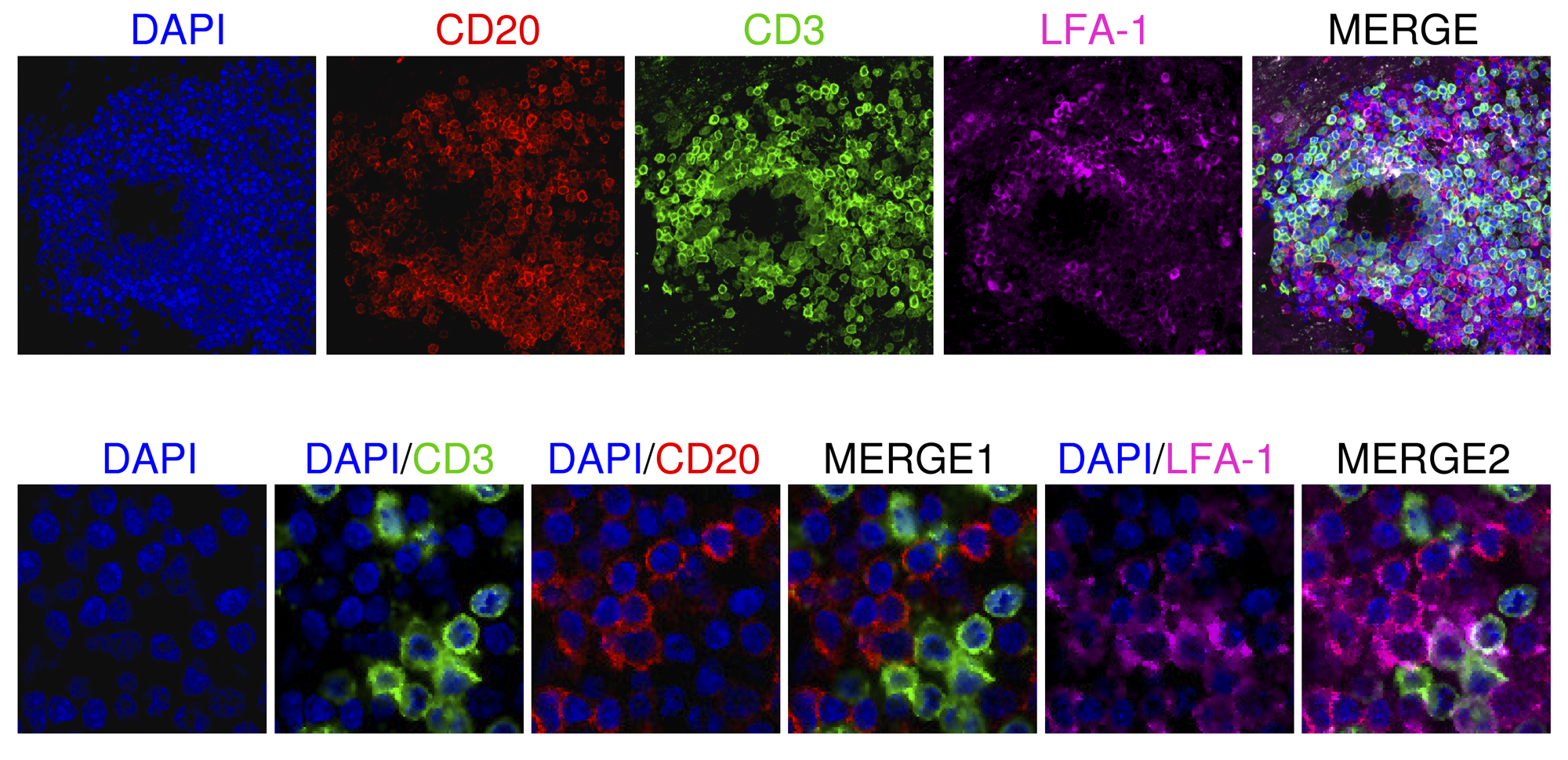

Supplement: Figure S5 — LFA-1 expression in lymphocytes in areas of infiltration after viral injection in monkey brain. Top panel shows over-expression of LFA-1 (magenta) in perivascular areas, where T-cells (green) and B cells (red) infiltrate the brain parenchyma. DAPI was used to stain the nuclei (blue). Bottom panel shows a detail of the area of infiltration of T and B lymphocytes co-localizing with LFA-1. (TIF) [file pone.0030762.s005.tif]

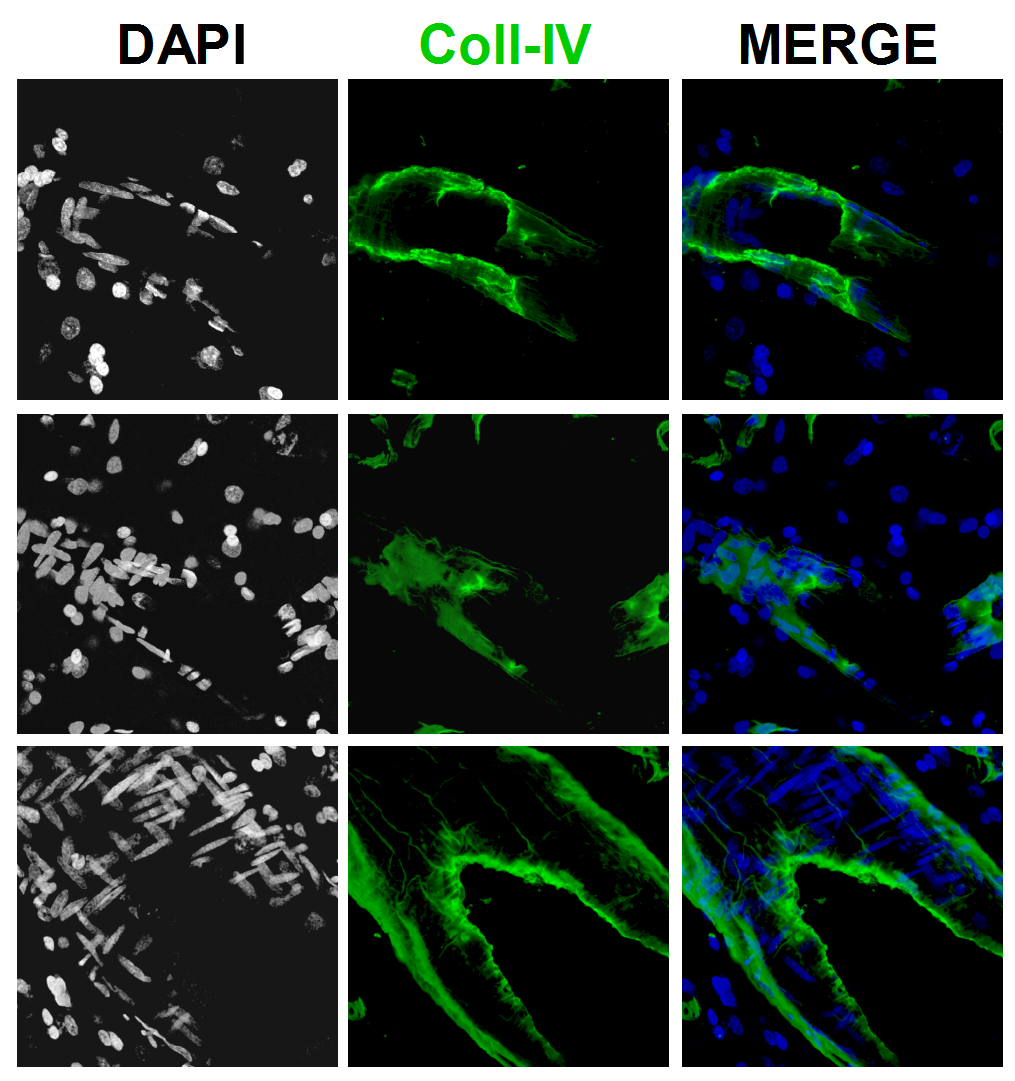

Supplement: Figure S6 — Staining of collagen-IV was performed to unequivocally delineate blood vessels shown in figure 3B . Confocal images show BVs in monkey brain sections stained with DAPI (white) and collagen-IV (green). In the merged images DAPI is shown in blue. (TIF) [file pone.0030762.s006.tif]

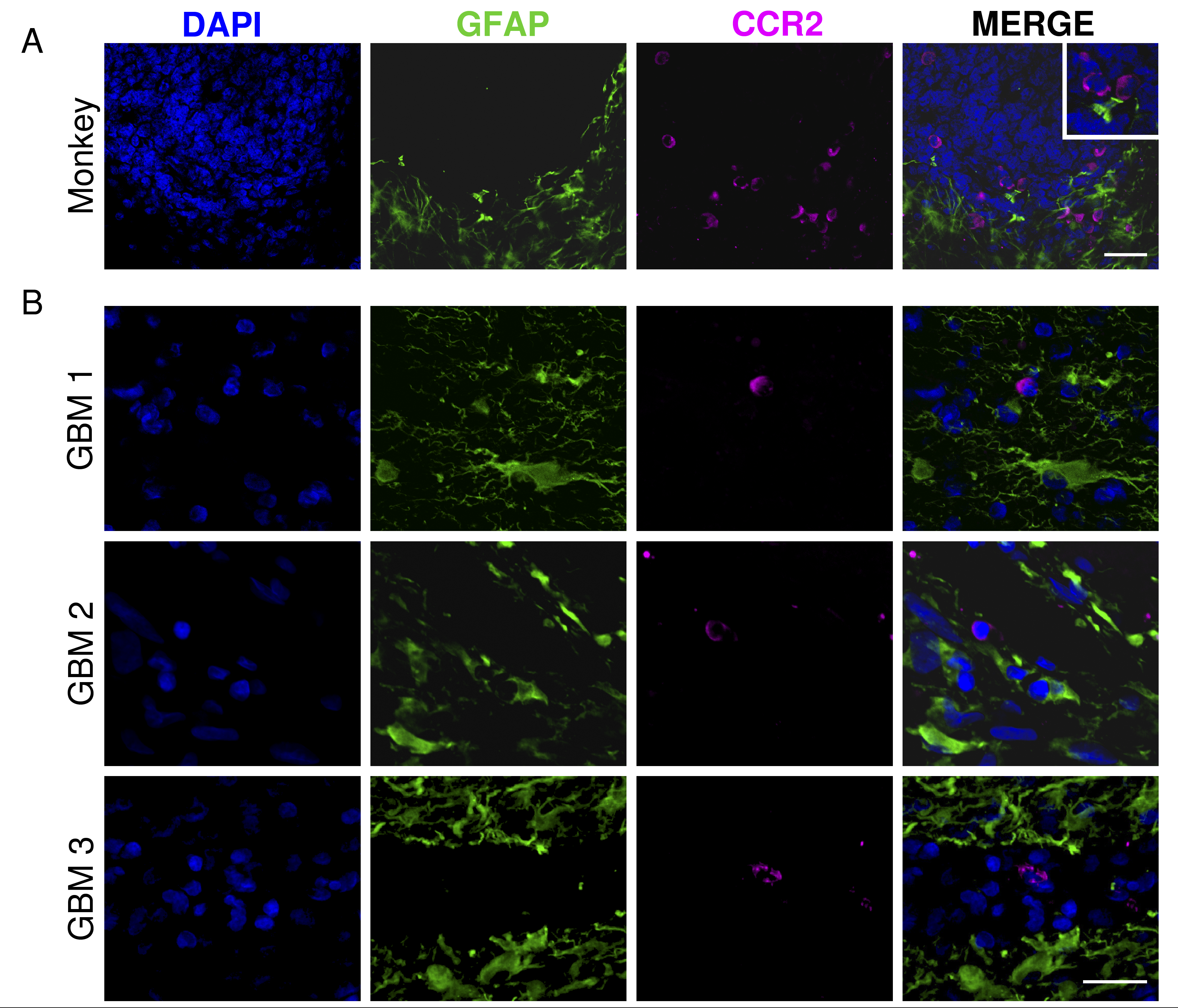

Supplement: Figure S7 — CCR2 is not expressed in GFAP+ astrocytes. Immuno-staining of CCR2 in monkey brain and samples of glioma combined with the astrocytic marker GFAP. (A) The areas of adenoviral injection in monkey brain show CCR2+ cells (magenta) and do not co-localize with GFAP marker (green). DAPI was used as a counterstaining (blue). Insert shows contact between a CCR2+ cell and a GFAP+ astrocyte. (B) Samples of glioma (GBM1, GBM2 and GBM3) show CCR2+ cells (magenta) and do not co-localize with GFAP+ astrocytes. DAPI was used as a counterstaining (blue). Scale bars: 50 µm. (TIF) [file pone.0030762.s007.tif]

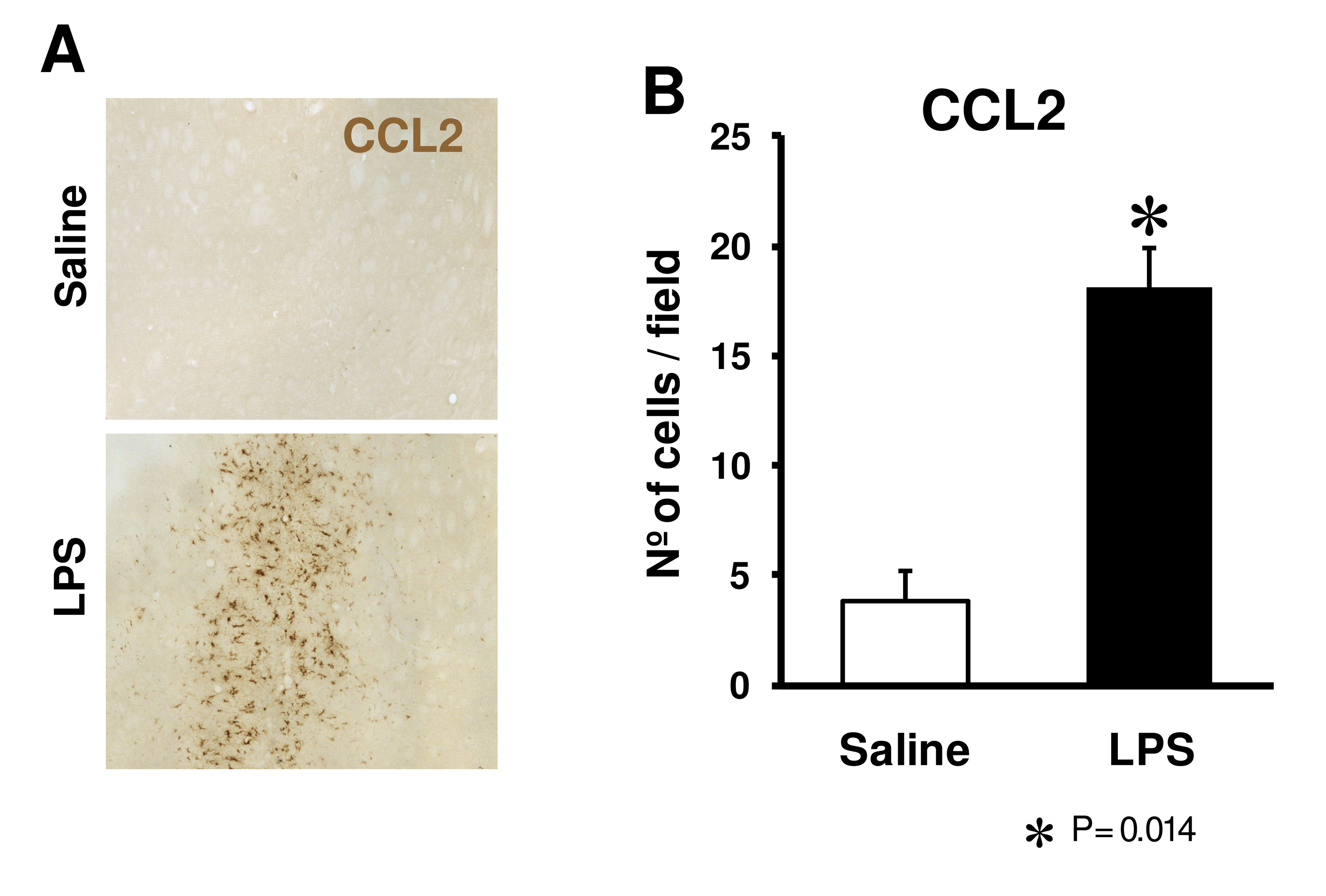

Supplement: Figure S8 — Increase in the number of CCL2+ cells in the mouse brain after LPS intrastriatal injection. (A) Representative picture of CCL2+ cells in the mouse striatum after LPS injection compared to a saline injection. (B) Quantification of CCL2+ cells in the injected striatum in a group of mice injected with saline compared with a group of mice injected with LPS. A dramatic increase of CCL2+ cells can be observed in the LPS injected mice. *p<0.05 Student t-test. (TIF) [file pone.0030762.s008.tif]
